# Heart Rate Variability Applications in Strength and Conditioning: A Narrative Review

**DOI:** 10.3390/jfmk9020093

**Published:** 2024-05-27

**Authors:** Jennifer S. Addleman, Nicholas S. Lackey, Justin A. DeBlauw, Alexander G. Hajduczok

**Affiliations:** 1 College of Osteopathic Medicine, Touro University California, Vallejo, CA 94592, USA; 2 Center for Applied Biobehavioral Sciences (CABS), Alliant International University, San Diego, CA 92131, USA; nslackey@gmail.com; 3 Department of Health and Human Physiological Sciences, Skidmore College, Saratoga Springs, NY 12866, USA; 4 Department of Cardiology, Thomas Jefferson University Hospital, Philadelphia, PA 19107, USA; alexander.hajduczok@jefferson.edu

**Keywords:** HRV-guided training, overtraining, training adaptations, training prescription

## Abstract

Heart rate variability (HRV) is defined as the fluctuation of time intervals between adjacent heartbeats and is commonly used as a surrogate measure of autonomic function. HRV has become an increasingly measured variable by wearable technology for use in fitness and sport applications. However, with its increased use, a gap has arisen between the research and the application of this technology in strength and conditioning. The goal of this narrative literature review is to discuss current evidence and propose preliminary guidelines regarding the application of HRV in strength and conditioning. A literature review was conducted searching for HRV and strength and conditioning, aiming to focus on studies with time-domain measurements. Studies suggest that HRV is a helpful metric to assess training status, adaptability, and recovery after a training program. Although reduced HRV may be a sign of overreaching and/or overtraining syndrome, it may not be a sensitive marker in aerobic-trained athletes and therefore has different utilities for different athletic populations. There is likely utility to HRV-guided programming compared to predefined programming in several types of training. Evidence-based preliminary guidelines for the application of HRV in strength and conditioning are discussed. This is an evolving area of research, and more data are needed to evaluate the best practices for applying HRV in strength and conditioning.

## 1. Introduction

Sport and exercise scientists are increasing their efforts to quantify and monitor athletes’ psychophysiological loads (e.g., training, psychological, and environmental) to optimize training adaptations. A promising inexpensive, time-efficient, and non-invasive method for monitoring athlete load is heart rate variability (HRV) [1,2]. HRV is defined as the fluctuation of time intervals between adjacent heartbeats (R-R intervals or inter-beat intervals (IBIs)) [1,3,4]. HRV is typically used as a surrogate measure of the autonomic balance of the sympathetic and parasympathetic nervous systems [1,3,4,5,6]. A normal heart has fluctuations in IBIs both at its baseline and in response to stimuli [4,5]. Although a core aspect of cardiac regulation is the pacemaker cells of the sinoatrial node, the heart rhythm is modulated by the autonomic nervous system (ANS) which receives feedback from baroreceptors and chemoreceptors from multiple blood vessels, information which is integrated in the medulla [5]. Given the regulatory role of the ANS as well as other top-down cortical processes augmenting the ANS response, HRV has been shown to be a helpful indicator of overall health of an individual [5,6]. A more in-depth review of the physiological mechanisms of HRV can be found in the work of Shaffer and colleagues [5]. While HRV has been measured and used in research applications as early as the 1970s, HRV has become an increasingly measured variable by wearable technology for use in fitness and sport applications [1,3,4].

HRV has shown promise as a tool for athlete monitoring and fitness or sport program modulation [7,8]. Additionally, HRV has become more accessible for sports science and sports performance professionals through the use of wearable devices. It is becoming increasingly important for strength and conditioning coaches, sports scientists, and sports medicine experts to be knowledgeable about how HRV should and should not be applied in their respective scopes of practice. While other reviews have evaluated the application of HRV in sports physiology [1,3,9], new data in the last 5–10 years prompt further comprehensive discussion. Lundstrom and colleagues discussed HRV’s application in endurance athletes and provided some preliminary recommendations for the literature [2]. While this is clearly beneficial, this may not fully reflect the training of athletes in which they incorporate both resistance and endurance training to maximize performance outcomes. Therefore, it is important to synthesize the current research on (a) the effects of resistance training on HRV, (b) the ability of HRV to assess physiological stress in response to resistance training, and (c) the use of HRV-guided resistance training. Furthermore, there is a lack of research connecting the effective utilization of HRV to guide training, specifically with strength training or combined modalities of strength and conditioning. No prior review has included multiple disciplines within strength and conditioning to create guidelines for the implementation of HRV. We provide preliminary guidelines to push HRV research forward and increase the standardized implementation of HRV in sports and fitness. **Primary Aim:** The current review proposes evidenced-based preliminary guidelines for the implementation of HRV in the practice of strength and conditioning and the expansion of the associated HRV literature.

## 2. Methods

A literature review was conducted to address and contextualize the current state of time-domain measures of HRV in strength and conditioning. PubMed and Scopus databases were utilized with the keywords “heart rate variability”, “strength”, “conditioning”, “overreaching”, “overtraining”, and “HRV-guided training”. Inclusion criteria included original research and review papers that discussed HRV, specifically time-domain-based measures, and its applications in the practice of strength and conditioning. Exclusion criteria included case reports and papers that did not include time-domain-based measures. 

## 3. Heart Rate Variability Measurement

HRV is regulated by the ANS. The standard methods of analysis of HRV fall into the time and frequency domains [3,4]. Time-domain metrics assess the difference between normal R-R intervals (NN), excluding ectopic beats. The time domain has been preferred in the field of strength and conditioning due to its stability and ability to be defined in short durations [10]. The most common time-domain units of measurement include the root mean square of the differences in successive R-R intervals (RMSSD), the standard deviation of NN intervals (SDNN), and the percentage of normal R-R intervals that differ by 50 ms (pNN50) [1,4]. The RMSSD time domain is commonly log-transformed for ease of interpretation by users [4]. RMSSD is preferred because it is more sensitive and less affected by respiratory rate, heart rate, or recording duration [1]. Other time-domain units, such as SDNN, must be standardized to a certain recording length to control for variance of HRV. This is very important with the increasing number of devices in the market that use various forms of HRV as metrics, as they cannot always be directly compared [11]. Several methodological approaches have been used to assess HRV including body position (i.e., supine, seated, and standing) and recording duration (i.e., 1, 5, and 7 min). A 5 min recording duration is recommended in the clinical setting [4], while ultra-short readings (i.e., 1 min) have been used with athletes [12], given their time constraints. Regardless of the position or measurement duration, consistency in measurement time and methodology is of foremost importance.

Frequency-domain methods allow for the distinction between high-frequency (HF) and low-frequency (LF) components. HF components (between 0.14 and 0.40 Hz) reflect the activity of the parasympathetic nervous system, while LF components (between 0.04 and 0.15 Hz) are generally accepted to reflect the activity of the sympathetic nervous system during longer recordings [10]. Examples of HF include respiration, and therefore respiratory sinus arrhythmia (this is how the respiratory rate can be back-calculated from the heart rate) are governed by vagal (parasympathetic) tones. LF signals that represent sympathetic tone include baroreflex activity and thermoregulation [13], and these require interpretive caution during short-term recordings (less than 24 h recordings) [5]. The ratio of HF/LF can assess the relationship between sympathetic and parasympathetic activity. Notably, frequency-domain measurements are confounded by respiration, which must be accounted for during the standardization of these metrics [14]. Frequency-domain measures are typically utilized to look at autonomic balance (parasympathetic withdrawal and/or sympathetic dominance). However, this has yet to be widely utilized in the strength and conditioning literature. 

**Figure 1:**
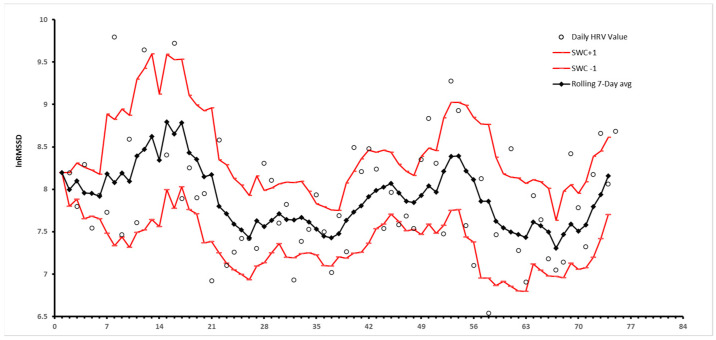
Visualization of a rolling 7-day average along with rolling smallest worthwhile changes (SWCs) visualized from unpublished data.

Importantly, HRV has no standardized range as of the current research, meaning there is no agreed upon normal value [4]. Normative ranges exist [15] but cannot be applied with cutoff values for ease of interpretability, especially with an athlete population. Therefore, the terms “higher” or “lower” are all relative to an individual’s baseline HRV. A high HRV relative to baseline represents a healthy, flexible autonomic system and improved adaptability and recovery in response to a changing environment or stressor, such as travel or exercise [1,3,16]. A low HRV relative to baseline represents an imbalanced autonomic function, typically due to a withdrawal of the parasympathetic system, resulting in a sympathetic-dominant state associated with increased inflammation and a greater potential for a cardiac event [1,3,17]. Regardless of high or low HRV, stability of HRV around an individual’s baseline within the smallest worthwhile change window (SWC) is typically considered a long-term goal of training. An SWC has previously been defined as ±0.5 or 1 standard deviations from an individual’s chosen HRV metric [18,19,20,21] (Figure 1).

### Measurement Considerations

The gold standard of measurement of HRV is through an electrocardiogram (ECG) [3,4]. However, many commercially available wearable devices measure HRV through photoplethysmography (PPG) [22] and heart rate sensors. PPG measurements can be trusted when a healthy individual is at rest [23], but physical or mental stress causes HRV metrics to meaningfully disagree with each other when comparing PPG sensors to the gold-standard ECG [22]. PPG may also become unreliable at heart rates greater than 160 BPM and with significant motion (particularly with wrist-worn devices), making interpretation during difficult workouts have limited utility. Furthermore, evidence exists showing that PPG accuracy is dependent on skin tone, with darker-skinned individuals having larger variabilities than lighter-skinned individuals [24]. Therefore, utilizing PPG for HRV monitoring in strength and conditioning should only be applied if utilization of ECG or heart rate sensors is impractical. Whether choosing to use PPG- or ECG-based devices, practitioners should prioritize internal consistency by utilizing the same device for all measurements.

Measurements of HRV are typically performed at rest, post-exercise, or continuously throughout the day or during exercise. Furthermore, HRV can be measured in various positions, including supine, seated, standing, or with orthostatic testing. Given that autonomic control of the cardiovascular system is moderated by several inputs detecting physiological changes [1], contextual factors such as period length, detection method, sampling frequency, removal of artifacts, body positioning, and respiration should be controlled when monitoring HRV, regardless of the method or metrics used [4]. Most importantly, HRV metrics provide limited utility during exercise given the sympathetic activation and parasympathetic withdrawal, preventing the intended measurement of autonomic balance. Therefore, most commercially available wearable devices monitor HRV during slow-wave (deep) sleep to minimize noise in the signal that is common when awake and moving [4]. Other wearable devices measure HRV immediately upon waking, standardizing the HRV measurement to exclude external stimuli (i.e., activities that would increase or decrease heart rate) without requiring devices to estimate sleep phases [4]. The key feature is standardization in the methodology of HRV measurement for each device, so it is internally consistent for the individual, and addressing the physiological or clinical question that is being investigated. A full discussion of the benefits and deficits of various HRV measurement techniques is outside of the current review’s scope but should be considered in future work.

HRV is non-invasive, easy to measure, and can be measured daily without physical exertion. Therefore, it can be applied widely and cheaply. Other physiologic measures of physical fitness, such as VO2 max or maximum strength testing, require experienced practitioners and additional equipment, and are unreasonable to be used for testing on a normative basis. HRV should be considered in addition to the other established performance metrics rather than in isolation [1].

## 4. Heart Rate Variability and Training Adaptations

### 4.1. Aerobic/Endurance-Trained Athletes

Several studies have identified that night-time RMSSD can significantly increase following training programs [25,26] in endurance athletes, including runners [26,27,28], cyclists [29], swimmers [30,31], and endurance walkers [32]. Furthermore, RMSSD has been correlated with an increase in training adaptations such as VO2 max, max running velocity, and timed-trial performance in this population [26,27]. There is also evidence that increased baseline HRV may be a reliable marker of training status or training age and that a delayed return to baseline HRV may be associated with reduced training age and/or adaptability in response to training [28,33].

### 4.2. Strength/Resistance-Trained Athletes

HRV measures and their associations with training adaptations in strength and high-intensity interval training have not been studied extensively. However, there is likely a correlation between HRV and adaptations to strength-based training [34]. No studies were found that investigated the association of HRV with maximum strength gains, rate of force development, critical torque, critical power, or cross-sectional area after a training program. Furthermore, no studies were found that investigated the changes in HRV associated with different forms of resistance training, such as Olympic weightlifting, powerlifting, and multimodal resistance training, or different resistance training intensities or durations. Given the limited research discussing training adaptations specific to resistance training and their connection to HRV, HRV should be interpreted on an individual basis in strength-based athletes [35]. 

### 4.3. Untrained or Moderately Trained Individuals

Independent of body mass index, waist circumference, and body fat percentage, individuals of the same age exhibited a significant positive relationship between self-reported physical activity during a training program and HRV [36,37,38]. This suggests that physical activity of any type may be associated with an increased baseline HRV. It is unclear whether HRV is associated with VO2 max in untrained or moderately trained individuals [39]. No studies were found that investigated the association of HRV with maximum running velocity, maximum strength gains, or cross-sectional area in untrained or moderately trained individuals.

## 5. HRV and Overreaching/Overtraining

### 5.1. Aerobic/Endurance-Trained Athletes

There is conflicting evidence regarding how HRV is affected by overreaching and/or overtraining syndrome in endurance athletes. As was previously discussed, trained endurance athletes typically experience a more rapid return to baseline HRV associated with their training age and performance [28,33]. Conversely, overtrained endurance athletes can experience HRV instability outside of the SWC similar to that of untrained individuals who are not overtrained, suggesting that overtrained athletes’ HRV responses to exercise are blunted compared to their baselines [40,41,42]. However, several studies have not found significant changes in highly trained endurance athletes’ HRVs after an overload training period, or even identified a paradoxical increase in HRV in overtrained athletes [27,43,44,45]. Evaluating rolling 7-day averages of an individual’s HRV compared to daily values may provide a more meaningful context on HRV changes and overtraining compared to daily measurements or population norms [20]. 

Athletes of several other sports, including soccer [46], wrestling [47], football [48,49], and rowing [50,51], have demonstrated HRV instability associated with increased workload, which may signal a transition to overreaching and/or overtraining syndrome. These findings may vary depending on the athletic capacity and training age of the study populations [48,49,50] as well as the sport type.

### 5.2. Strength/Resistance-Trained Athletes

In resistance-trained athletes, increasing resistance training load via increasing the volume and/or intensity results in a prolonged reduction in both HRV [52,53,54] and subsequent performance testing at 48 h post-training session [55,56] that may resolve only after a multi-day recovery period [54]. However, recovery time may vary on an individual and group level in strength-trained athletes [57], and HRV could normalize as quickly as 1 h after training [58]. Increased training load is also associated with a perturbation in biochemical markers related to fatigue, including testosterone, cortisol, growth hormone, prolactin, IGF-1, and creatine kinase at 48 h post-training session [55,56]. Furthermore, the combination of a reduced HRV and a high acute–chronic workload ratio may be associated with an increased risk of overuse injuries [59].

### 5.3. Untrained or Moderately Trained Individuals

Moderately active and untrained adults likely exhibit greater reductions in HRV when overtrained, suggesting that their reduced training age may put them at greater risk for overreaching and/or overtraining syndrome [40]. Endurance exercise sessions of longer durations, i.e., 90 min, may result in a perturbation of HRV in untrained or moderately trained individuals that is not seen in aerobically-trained athletic populations or after endurance exercise sessions of 30 or 60 min [60]. This suggests that untrained individuals may be able to minimize an HRV reduction in response to training with endurance exercise sessions which have durations of 30 or 60 min. Furthermore, continued consistent training in this population may improve their ability to respond to training load at longer durations, such as 90 min [60].

## 6. Heart Rate Variability-Guided Programming

HRV-guided programming in various fitness and sport applications has been investigated and may be used to make training decisions by many athletes and coaches [61]. Typically, an athlete’s baseline resting HRV and SWC are established first [21,61,62]. When an athlete’s HRV is inside their SWC, they train as normal with a moderate to high intensity training session [61,62]. When the athlete’s HRV is outside their SWC, training is modified to include either a low intensity training session, a prescribed rest day, or the addition of activities to promote recovery (mobility, stretching, etc.) [61,62]. This is in contrast to a predefined training model, which includes varying high, moderate, and low intensity training days for an established training block, but without considering HRV or other recovery metrics. This has become increasingly popular as its use has shown to induce superior training adaptations compared to predetermined training in several fitness and sport applications as discussed in the subsequent sections. Furthermore, HRV has been used to assess trainability [7,8] and injury susceptibility [63,64] in competitive athletes. The use of HRV-guided training allows for a more individualized approach to training as well as adaptation to an individuals’ specific physiology and environmental conditions.

### 6.1. Aerobic/Endurance-Trained Athletes

Endurance training utilizing HRV-guided programming is likely the most studied. HRV-guided programming for endurance athletes is associated with significant increases in maximum running velocity, VO2 max, VO2 at the first and second ventilatory thresholds, peak power output, HRV, and serum testosterone levels compared to predefined or block periodized groups [18,65,66,67]. This association has been identified in running [18,62], cross-country skiing [68], and cycling [66]. It is likely that HRV-guided training has utility in elite or highly trained endurance athletes [69]. A recent review of applications of HRV monitoring in endurance athletes provides a comprehensive overview of the current research for this population and suggests that daily HRV monitoring and HRV-guided training has promising applications for endurance athletes [2].

### 6.2. Strength/Resistance-Trained Athletes

No difference has yet been found between HRV responses after various resistance training methods, including traditional, paired set, superset, circuit, and multiple set training [70,71], suggesting that the specific training method should be selected based on training goals rather than changes in HRV. High acute–chronic workload ratios in training were tolerated better by CrossFit^TM^ athletes with less risk of overuse problems when the rolling 7-day average HRV was normal or high compared to when the rolling 7-day average HRV was low [59], suggesting a relationship between HRV and the tolerance of high-intensity training load in multimodal training. Different athletes have been found to experience varying time frames of recovery for HRV following intense resistance training at both the group and individual levels [57], implying that HRV-guided programming may be a beneficial tool to modify intensity depending on an athlete’s day-to-day recovery. However, there has not been an identifiable difference between an individual’s muscle hypertrophy and strength responses after HRV-guided programming compared to fixed programming [72].

### 6.3. Untrained or Moderately Trained Individuals

Moderately active men and women, recreational endurance runners, and untrained women have experienced similar significant improvement in muscle hypertrophy, strength, VO2 max, body composition, and fitness with a lower training load in an HRV-guided training group compared to a predefined training group across different types of training [19,61,62,72,73]. Differential effects existed in which the HRV-guided training groups had increased performance over their standard comparison groups for endurance-style training [19,61,72] but did not have a significantly increased strength or hypertrophic response [73]. Most importantly, all groups did achieve improved or similar gains in overall performance with fewer high-intensity training days [19,61,62,72,73]. Further investigation into the sex differences of HRV-training is warranted given the variability in the current, limited literature [19], with some authors insinuating greater benefit for women over men [73]. There has not been a comparative study between the efficacy of HRV-guided training for strength training, endurance training, or combined training in untrained or moderately trained individuals. Given the current studies, variability in the implementation of HRV-guided training may be dependent on the modality of training for untrained or moderately trained individuals.

## 7. Factors Interacting with Heart Rate Variability

There are many factors that both influence an individual’s HRV baseline and are associated with perturbations from an individual’s baseline. Increased age [74]; male sex [74]; increased BMI [74]; poor sleep time and quality [75]; stress [9] and anticipation of a stressful event [76]; consumption of alcohol and/or nicotine [77,78]; dehydration [79]; acute sickness [80]; post-vaccination symptomatology [81]; acute and chronic pain [82,83,84]; concussion and post-concussion recovery [85,86,87]; travel or training camps [8,51]; medications including but not limited to beta-blockers [88], angiotensin-converting enzyme inhibitors [89], contraceptive medications [90], and antidepressants [91]; and many psychiatric and physiological diseases [91,92] can be associated with a reduced HRV. Weight loss in overweight and obese individuals is associated with an improved HRV from their original baseline [93]. Additionally, HRV often varies in a predictable pattern associated with the female menstrual cycle [94,95] and may be related to factors that influence training, such as sleep quality, stress, injury, motivation, and program enjoyment [96]. Due to the many factors that influence HRV, professionals interpreting HRV data should ensure they only act within their appropriate scope of practice. Strength coaches, physical therapists, athletic trainers, and sports scientists should ensure they refer to sports medicine physicians if they have any health concerns for specific individuals based on their HRV pattern or if athletes have an ongoing medical issue that may require additional monitoring. If concerns about mental health disorders or psychiatric diseases arise, referrals to sports psychologists or psychiatric professionals should be considered. HRV biofeedback is a potentially valuable tool in the management of stress and chronic disease and should only be performed by a licensed individual who is board-certified in the modality [9]. 

Given the possible effects of HRV, it is also important to be wary of excessive checking on the order of minutes to hours which may have a stress-inducing effect on athletes. This will arbitrarily have a decreasing effect on HRV, given the increase in anxiety and the corollary decrease in parasympathetic activation. It may be helpful to blind athletes to their daily HRV values to reduce anxiety, though many personal wearable devices make blinding difficult. It has been suggested that while HRV is sensitive to psychological stress in preparation for sporting events, it is not associated with reductions in performance [97]. Pragmatically, HRV is an index of overall autonomic flexibility, and the decrements in HRV associated with competition result from the natural reduction in parasympathetic activity, as seen in the HRV and stress literature [98]. However, further studies must explore delineations between abnormally anxious responses in athletes versus HRV decrements from competitions and other normative stressful events.

## 8. Preliminary Guidelines

In summary, our preliminary guidelines from the current literature for using HRV in the strength and conditioning setting are as follows:

1. In the scope of strength and conditioning, relative differences to an individual’s own baseline are the best interpretive level. Interpreting further may have limited use.
2. HRV is a helpful metric to assess training status, adaptability, and recovery of trained athletes in a variety of sports and fitness activities as well as untrained individuals. Coaches can utilize this information to evaluate an athlete’s preparedness for a difficult training day. Regardless of a high or low HRV, stability of HRV within the smallest worthwhile change window (SWC) is considered a long-term goal of training.
3. Barring all other contextual factors, decreasing HRV may be a sign of overreaching and/or overtraining syndrome. In aerobic athletes, HRV metrics may not be as sensitive to overreaching and/or overtraining syndrome and may be best utilized in addition to secondary markers. Further investigations are needed to fully understand the autonomic response to overreaching and overtraining syndrome and which measures are best to predict or identify these changes.
4. There is utility to HRV-guided programming compared to predefined programming in several sports and athletic activities, especially in training programs with an aerobic or endurance component.
5. There are many factors that influence an individual’s HRV, including but not limited to various physiological and psychological disorders. Professionals interpreting HRV data should ensure they only act within their appropriate scope of practice and refer to an appropriate healthcare professional as needed.

There is not enough research, therefore we provide these guidelines from the available evidence to increase the proliferation of rigorous HRV literature in strength and conditioning. Table 1 provides useful starting points for the implementation of HRV in strength and conditioning.

## 9. Limitations and Future Directions

Many of the aforementioned studies have small sample sizes that limit the applicability of the discussed information. However, the overall conclusions are still helpful, particularly when included in meta-analyses. Nonetheless, more research is needed to support and clarify the best practices for utilizing HRV in strength and conditioning. Many of these studies have limited generalizability in youth athletes, as most research was conducted with adult subjects. Further research should not only extend this ideology to all age groups but also to different modalities of exercise to ensure the applicability of these guidelines to strength and conditioning more broadly. Furthermore, strength and conditioning professionals should refer to individual studies for the methodology of how to specifically integrate HRV into their own coaching practice and approach each athlete on a case-by-case basis.

There are many other heart rate metrics that are heavily studied, including resting heart rate and heart rate recovery (HRR). HRR is the rate at which the heart rate decreases within the following minutes after high-intensity exercise that approaches maximum heart rate. Deciding on which heart rate metric to monitor in a strength and conditioning setting is debated heavily in the literature [99,100]. Discussion on the uses, strengths, and/or limitations of HRR and HRR versus HRV is outside of the scope of the current review.

Overall, HRV research in strength and conditioning is still in its infancy. Due to the limited sample sizes common with research studying specific athletes and the large variability in HRV measurement methodologies, there is substantial heterogeneity in the current literature. The guidelines discussed in this paper should serve as a guide to standardize future research with the hope for further studies, reviews, and meta-analyses to clarify the utility of HRV in various athletic populations. Further research should investigate the HRV response after both short-term and long-term training programs, and whether HRV is associated with specific training adaptations. While HRV has been studied more extensively in endurance athletes, there is a gap in the literature regarding its application in resistance-trained athletes. Future research should investigate if a stable or high HRV is associated with increased maximum strength gains, rate of force development, critical torque, critical power, or cross-sectional area after a training program compared to an unstable or low HRV. This should also include identifying the effects of exercise in different forms of resistance training on HRV, such as Olympic weightlifting, powerlifting, and multimodal resistance training. Some preliminary studies are investigating the effects of non-athletic stressors (i.e., travel [101], illness [8,51], and sleep [102]) on athletic populations and their possible impacts on subsequent performance. Further investigations are warranted to identify the sensitivity of HRV in detecting and predicting overreaching. Additional research should investigate the applicability of HRV in different sports and further delineate which sports are best suited for HRV monitoring around training and/or competitions. Given the increased public access to HRV data from the use of wearable devices in untrained or moderately trained individuals, it is also important to establish its utility for this population.

The long-term physiological changes that may be related to HRV-guided training have yet to be delineated. Such understandings are required because the associated changes in HRV with HRV-guided training may be small [103], thus requiring large samples, which are currently uncommon and unrealistic in athletic populations. Given that the goal of HRV-guided training is to increase training adaptability, it is important to note that implementing HRV-guided training can result in small gains in training efficiency that may still significantly increase elite athletes’ performances and improve outcomes. Understanding such physiological processes in relation to HRV would increase the understanding of training adaptations and overtraining in athletes. The magnitude of these physiological changes may be different in untrained or moderately trained individuals, requiring subsequent validation and different interpretation.

## 10. Conclusions

Heart rate variability is a helpful metric in strength and conditioning to assess athletic performance and programs in a variety of sports and fitness activities. Different athletic populations have different utilities for HRV to identify or predict overreaching and/or overtraining syndrome. There is likely utility to HRV-guided programming compared to predefined programming in several sports and athletic activities. This is an evolving area of research, and more data are needed to fully evaluate the best practices for applying HRV in strength and conditioning.

## Figures and Tables

**Table 1:** Guidelines for Implementation in Strength and Conditioning.

Guidelines for Implementation in Strength and Conditioning	
Do’s	Don’ts
Record for a consistent duration of measurement, with best practice being at least 5 min Measure at the same time of day Measure at rest Measure with the same body positioning Use the same device settings (i.e., sampling frequency, period length, detection method, artifact removal) Know what data your app is giving you (i.e., time of measurement, arbitrary measurement vs HRV value) Track general trends with smallest worthwhile change (SWC) window using 7-day rolling averages Consider the stability of HRV within SWC rather than high or low HRV as the long-term goal of training, barring other contextual factors that affect HRV (i.e., stress) Consider the other stimuli that can affect HRV outside of training and sports Consider utilizing HRV-guided training depending on the training context	Apply population-based normalized ranges at the individual level with cut-off values Measure immediately after a “stimulating” event: exercise and high-stress life events Over-interpret small changes in HRV Change the entire training regimen over small HRV changes Rely on HRV measurements in isolation to detect overreaching and/or overtraining syndrome Act outside of the appropriate scope of practice
